# Delivery room intubation and severe intraventricular hemorrhage in extremely preterm infants without low Apgar scores: A Japanese retrospective cohort study

**DOI:** 10.1038/s41598-023-41010-x

**Published:** 2023-09-11

**Authors:** Kei Tamai, Naomi Matsumoto, Takashi Yorifuji, Akihito Takeuchi, Makoto Nakamura, Kazue Nakamura, Misao Kageyama, The Neonatal Research Network of Japan

**Affiliations:** 1 Division of Neonatology, Okayama Medical Center, National Hospital Organization, 1711-1 Tamasu, Kita-Ku, Okayama, 701-1192 Japan; 2 Department of Epidemiology, Okayama University Graduate School of Medicine, Dentistry and Pharmaceutical Sciences, Okayama, Japan

**Keywords:** Paediatric research, Epidemiology

## Abstract

The purpose of this study was to assess the associations between delivery room intubation (DRI) and severe intraventricular hemorrhage (IVH), as well as other neonatal outcomes, among extremely preterm infants without low Apgar scores using data from a large-scale neonatal registry data in Japan. We analyzed data for infants born at 24–27 gestational weeks between 2003 and 2019 in Japan using robust Poisson regression. Infants with low Apgar scores (≤ 1 at 1 min or ≤ 3 at 5 min) were excluded. The primary outcome was severe IVH. Secondary outcomes were other neonatal morbidities and mortality. The full cohort included 16,081 infants (intubation cohort, 13,367; no intubation cohort, 2714). The rate of DRI increased over time (78.6%, 2003–2008; 83.4%, 2009–2014; 87.8%, 2015–2019), while the rate of severe IVH decreased (7.1%, 2003–2008; 5.7%, 2009–2014; 5.3%, 2015–2019). Infants with DRI had a higher risk of severe IVH than those without DRI (6.8% vs. 2.3%; adjusted risk ratio, 1.86; 95% confidence interval, 1.33–2.58). The results did not change substantially when stratified by gestational age. Despite conflicting changes over time in DRI and severe IVH, DRI was associated with an increased risk of severe IVH among extremely preterm infants in Japan.

## Introduction

Intraventricular hemorrhage (IVH) is a major short-term complication of prematurity that can result in long-term adverse outcomes^1^. Although the occurrence of IVH declined since the 1980s, the incidence of IVH remained relatively high in the 2000s^2, 3^. The National Institute of Child Health and Human Development Neonatal Research Network reported that the incidence of severe IVH in infants born extremely preterm (EPT; i.e., 22–28 gestational weeks) was 14.3% in 2013–2018^4^. IVH remains a major problem for premature infants in neonatal intensive care units (NICUs).

The causes of IVH are multifactorial. Reported risk factors for IVH include vaginal delivery, low Apgar score, respiratory distress syndrome (RDS), pneumothorax, hypoxia, hypercapnia, seizures, patent ductus arteriosus (PDA), thrombocytopenia, and infection^1^. Several studies showed that postnatal ventilatory management, including delivery room intubation (DRI), which has an important role in neonatal care, was associated with IVH among infants born very preterm (i.e., < 32 gestational weeks)^5, 6^. The Committee on the Fetus and Newborn of the American Academy of Pediatrics recommended the initial use of continuous positive airway pressure in the delivery room rather than intubation^7^. While rates of DRI in the US and other countries have declined in recent decades (e.g., in the US, 22–28 weeks of gestation, 80% in 1993 to 65% in 2012)^5, 8–10^, the rate of DRI among infants born at 22–32 weeks of gestation in Japan increased from 50.1 to 63.8% between 2003 and 2012^11^. Thus, EPT infants in Japan tended to receive more invasive respiratory management in recent days, but had a lower incidence of severe IVH than those in other high-income countries^12–14^. These findings raise a contradiction in previous studies suggesting an association between invasive respiratory management and IVH. However, few large-scale cohort studies have investigated associations between DRI and neonatal morbidities, including severe IVH among EPT infants, especially in countries such as Japan that favor invasive respiratory management.

The present study examined associations between DRI and severe IVH and other neonatal outcomes among EPT infants without low Apgar scores using large-scale population-based neonatal registry data from Japan.

## Methods

### Study cohort

This study was a retrospective cohort study based on the data from the Neonatal Research Network of Japan (NRNJ) which created a network database of very-low-birth-weight infants (i.e., birth weight ≤ 1,500 g) across perinatal medical centers in Japan. Overall, 125 institutions (60% tertiary centers) were registered in the NRNJ in 2018. The NRNJ collected perinatal data for very-low-birth-weight infants during their hospitalization in NICUs and follow-up visits. Written informed consent to use the data for future neonatal care research was obtained from all patients at each institution. EPT infants born between 2003 and 2019 were included in the present study. We excluded infants with out-of-hospital birth, congenital anomalies, death in the delivery room, no data on intubation status in the delivery room, or incomplete severe IVH data. We also excluded infants with a gestational age of 22–23 weeks or with severe asphyxia because DRI appears to be unavoidable in such infants. Referring to previous studies, the cut-off points for severe asphyxia for EPT infants were defined as Apgar scores of ≤ 1 point and ≤ 3 points at 1 and 5 min, respectively^15–17^. This study was conducted in accordance with the Declaration of Helsinki and approved by the central internal review board at Osaka Women’s and Children’s Hospital (#1104-3).

### Exposure

The exposure variable was DRI obtained from the NRNJ database. Data on details of DRI were not available, such as the indication for DRI, number of attempts, experience of operators, or use of premedication.

### Outcomes

IVH was classified according to the grading scale reported by Papile et al^18^. Severe IVH was defined as grade 3 or higher. Japanese neonatologists frequently perform brain ultrasounds of EPT infants with a high risk of IVH during the initial 72 h after birth^12^. Periventricular leukomalacia was diagnosed by head ultrasound or magnetic resonance imaging. Chronic lung disease (CLD) was defined as a condition requiring supplemental oxygen or positive pressure ventilation at 36 weeks of postmenstrual age. PDA was diagnosed on the basis of physical findings and echocardiogram, and each medical institution made its own decision on the indication of surgery for hemodynamically significant PDA. Necrotizing enterocolitis was defined according to radiographic or surgical findings and the criteria used by Bell et al. (stage 2 or higher)^19^. The primary outcome was severe IVH (grade 3 or higher). Secondary outcomes were pre-discharge mortality and other neonatal morbidities such as IVH of any grade (grades 1–4), periventricular leukomalacia, CLD, PDA requiring surgery, and necrotizing enterocolitis.

### Definitions of confounders

Gestational age was determined from findings of fetal ultrasound during the first trimester and the date of the last menstrual period. Small for gestational age was defined as a birth weight below the 10th percentile for gestational age on the basis of Japanese standard neonatal anthropometric charts^20^. Non-reassuring fetal status was defined as a fetal condition with abnormal fetal heart rate monitoring (e.g., absent fetal heart rate baseline variability, recurrent variable decelerations, prolonged decelerations, bradycardia, and sinusoidal pattern)^21^. Premature rupture of membranes was defined as rupture of the amniotic sac before labor began. Clinical chorioamnionitis was diagnosed on the basis of clinical signs, such as maternal fever (≥ 38.0 °C), leukocytosis (≥ 15,000/μl), tachycardia (heart rate ≥ 100 beats per minute), uterine tenderness, and the presence of purulent fluid from the cervical os^22^. Hypertensive disorder of pregnancy was defined as hypertension (systolic blood pressure ≥ 140 mmHg and/or diastolic blood pressure ≥ 90 mmHg) during pregnancy^23^. Antenatal steroids were defined as the administration of any dose of corticosteroids given to the mother before delivery. Placental transfusion was defined as the transfer of residual placental blood to the newborn at birth, without specifying whether umbilical cord milking or delayed cord clamping was utilized. In Japan, umbilical cord milking is commonly performed in NICUs for extremely preterm infants, according to the guidelines set by the Neonatal Cardiopulmonary Resuscitation Program in Japan^24^. RDS was diagnosed by clinical and radiographic findings^25^.

### Statistical analyses

To evaluate the current status of NICUs in Japan, we assessed trends from 2003 to 2019 in treatment and neonatal morbidities among EPT infants during NICU hospitalization. We then compared baseline characteristics between infants who were intubated in the delivery room and those who were not. The differences in the proportion between the group with and without DRI were tested by using the χ2 test. Subsequently, we conducted Poisson regression models with robust variance estimators to investigate the associations between DRI and neonatal morbidities and mortality. In addition, we performed a stratified analysis to examine the impact of DRI on neonatal morbidities and mortality stratified by gestational age category (i.e., 24–25 and 26–27 gestational weeks). We estimated the risk ratio (RR) and 95% confidence interval (CI) for the main outcomes using the infants who were not intubated in the delivery room as a reference, after adjusting for child and maternal factors.

We selected potential confounders on the basis of previous studies^5, 6, 26^. Child factors included gestational age (24, 25, 26, and 27 gestational weeks; categorical), birth weight (< 500, 500–749, 750–999, and ≥ 1,000 g; categorical), small for gestational age (dichotomous), sex (dichotomous), multiple births (dichotomous), facility-level at birth (tertiary, secondary, and others; categorical), birth year (2003–2008, 2009–2014, and 2015–2019; categorical), Apgar scores at 1 and 5 min (0–3, 4–6, and 7–10; categorical), placental transfusion (dichotomous), and RDS (dichotomous). Maternal factors included maternal age (< 25, 25–29, 30–34, and ≥ 35 years; categorical), antenatal steroids (dichotomous), cesarean section (dichotomous), non-reassuring fetal status (dichotomous), premature rupture of membranes (dichotomous), clinical chorioamnionitis (dichotomous), and hypertensive disorder of pregnancy (dichotomous).

In the sensitivity analysis, we examined the association between DRI and composite outcome, death or severe IVH. Stata SE version 16 statistical software (StataCorp., College Station, TX, US) was used for all analyses. P values less than 0.05 were considered significant.

## Results

**Figure 1:**
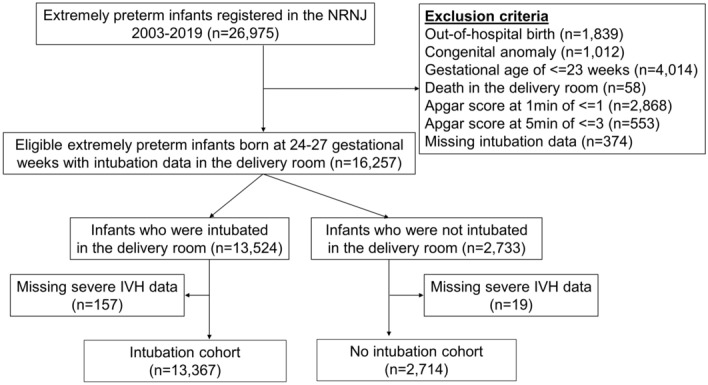
Study flow chart. IVH, intraventricular hemorrhage; NRNJ, Neonatal Research Network in Japan.

Between 2003 and 2019, 26,975 EPT infants were registered in the NRNJ. Of these infants, 10,718 EPT infants were excluded because of out-of-hospital birth (n = 1,839), congenital anomaly (n = 1,012), gestational age ≤ 23 weeks (n = 4,014), delivery room death (n = 58), Apgar score at 1 min of ≤ 1 (n = 2,868), Apgar score at 5 min of ≤ 3 (n = 553), or no data on intubation status in the delivery room (n = 374). Of the remaining 16,257 EPT infants, 176 EPT infants had incomplete severe IVH data. Finally, 16,081 EPT infants were included in our study population (Fig. 1).

Table 1 and Supplementary Table S1 online show the trends from 2003 to 2019 in the treatment and morbidities of EPT infants during NICU hospitalization. The frequency of DRI, antenatal steroids, cesarean section, and surfactant administration during hospitalization showed upward trends. Regarding neonatal morbidities, the incidence rates of severe IVH and mortality showed downward trends, whereas the incidence of RDS and CLD showed upward trends, and the incidence of any grade IVH remained unchanged.

**Table 1:** Trends in treatment and morbidities of eligible extremely preterm infants (24–27 weeks of gestational age) in the Neonatal Research Network of Japan.

	Birth Year					
	2003–2008		2009–2014		2015–2019	
	(n = 4819)		(n = 6986)		(n = 4276)	
Delivery room intubation	3788	(78.6)	5826	(83.4)	3753	(87.8)
24–25 weeks of gestational age	1728/1930	(89.5)	2462/2710	(90.8)	1607/1698	(94.6)
26–27 weeks of gestational age	2060/2889	(71.3)	3364/4276	(78.7)	2146/2578	(83.2)
Antenatal steroids	2291	(47.5)	4410	(64.0)	3083	(73.0)
Cesarean section	3655	(75.8)	5598	(80.3)	3551	(83.6)
Respiratory distress syndrome	3415	(70.9)	5373	(77.5)	3482	(81.8)
Surfactant administration during hospitalization	3457	(71.7)	5526	(80.0)	3614	(85.6)
Mortality	421	(8.8)	356	(5.1)	157	(3.7)
Severe IVH (grade 3–4)	344	(7.1)	397	(5.7)	228	(5.3)
IVH (any grade)	992	(20.6)	1283	(18.4)	825	(19.3)
Chronic lung disease	1327	(30.2)	2739	(42.9)	1874	(48.2)

Values are n (%). IVH, intraventricular hemorrhage.

Table 2 shows the baseline characteristics for eligible infants without low Apgar scores who were intubated in the delivery room and those who were not. Compared with EPT infants without DRI, those with DRI had a younger gestational age, lower birth weight, lower Apgar scores at 1 and 5 min, lower incidence of premature rupture of membranes, and higher incidence of small for gestational age, multiple births, placental transfusion, RDS, surfactant administration, cesarean section, non-reassuring fetal status, and clinical chorioamnionitis. The same information stratified by gestational age category (i.e., 24–25 and 26–27 gestational weeks) is shown in Supplementary Table S2 online, with similar trends as shown in Table 2 for both gestational age categories.

**Table 2:** Baseline characteristics. Values are n (%) unless otherwise indicated.

	Delivery room intubation				
	Intubation		No intubation		*P* value^a^
	(n = 13,367)		(n = 2714)		
Characteristics of infants					
Mean gestational age (SD), weeks	26.1	(1.1)	26.7	(1.0)	< 0.001
24–25 weeks	5797	(43.4)	541	(19.9)	
26–27 weeks	7570	(56.6)	2173	(80.1)	
Mean birth weight (SD), g	793	(188)	883	(187)	
< 500 g	817	(6.1)	53	(2.0)	< 0.001
500–749 g	4874	(36.5)	604	(22.3)	
750–999 g	5839	(43.7)	1325	(48.8)	
≥ 1,000 g	1837	(13.7)	732	(27.0)	
Small for gestational age	3007	(22.5)	477	(17.6)	< 0.001
Sex, male	7024	(52.6)	1446	(53.3)	0.479
Multiple births	2751	(20.6)	470	(17.3)	< 0.001
Facility level at birth					
Tertiary	11,209	(83.9)	2,396	(88.3)	< 0.001
Secondary	1945	(14.6)	302	(11.1)	
Others	213	(1.6)	16	(0.6)	
Birth year					
2003–2008	3788	(28.3)	1031	(38.0)	< 0.001
2009–2014	5826	(43.6)	1160	(42.7)	
2015–2019	3753	(28.1)	523	(19.3)	
Median Apgar score at 1 min (25–75%tile)	4	(3–6)	6	(5–8)	
2 to 3	4061	(30.5)	287	(10.8)	< 0.001
4 to 7	8486	(63.6)	1684	(63.6)	
8 to 10	789	(5.9)	675	(25.5)	
Median Apgar score at 5 min (25–75%tile)	7	(6–8)	8	(7–9)	
4 to 7	8250	(62.5)	772	(29.3)	< 0.001
8 to 10	4944	(37.5)	1859	(70.7)	
Placental transfusion	3993	(30.0)	493	(18.2)	< 0.001
Respiratory distress syndrome	10,796	(81.1)	1474	(54.5)	< 0.001
Surfactant administration during hospitalization	11,180	(84.4)	1417	(52.4)	< 0.001
Maternal characteristics					
Maternal age, years					
< 25	1365	(10.4)	282	(10.8)	0.693
25–29	3051	(23.2)	608	(23.4)	
30–34	4591	(34.9)	918	(35.3)	
≥ 35	4159	(31.6)	793	(30.5)	
Antenatal steroids	8102	(61.1)	1682	(62.6)	0.149
Cesarean section	10,807	(81.1)	1997	(73.8)	< 0.001
Non-reassuring fetal status	3135	(24.0)	494	(18.5)	< 0.001
Premature rupture of membranes	4993	(37.5)	1108	(41.0)	0.001
Clinical chorioamnionitis	3606	(27.6)	655	(25.1)	0.007
Hypertensive disorder of pregnancy	1740	(13.1)	317	(11.7)	0.054

^a^The differences in the proportion between the group with and without DRI were tested by using the χ2 test.

Table 3 shows the associations between DRI and neonatal morbidities and mortality among infants born at 24–27 gestational weeks without severe asphyxia. EPT infants who were intubated in the delivery room had a higher risk for severe IVH than those who were not intubated, after adjusting for all covariates (6.8% vs. 2.3%; RR, 1.86; 95% CI, 1.33–2.58). In addition, DRI was associated with higher risks for any grade IVH (RR, 1.64; 95% CI, 1.41–1.92), CLD (RR, 1.10; 95% CI, 1.02–1.18), and PDA requiring surgery (RR, 1.65; 95% CI, 1.34–2.02), compared with no DRI.

**Table 3:** Associations between delivery room intubation and neonatal morbidities and mortality among extremely preterm infants without severe asphyxia.

	Delivery room intubation			
	Intubation		No intubation	
	(n = 13,367)		(n = 2714)	
Severe IVH (grade 3–4)				
Case/N	906/13,367	(6.8)	63/2714	(2.3)
RR (95% CI)^a^	1.86	(1.33–2.58)	1	(Ref)
IVH (any grade)				
Case/N	2850/13,367	(21.3)	250/2714	(9.2)
RR (95% CI)^a^	1.64	(1.41–1.92)	1	(Ref)
Periventricular leukomalacia				
Case/N	510/13,299	(3.8)	86/2707	(3.2)
RR (95% CI)^a^	1.28	(0.94–1.75)	1	(Ref)
CLD				
Case/N	5201/12,128	(42.9)	739/2549	(29.0)
RR (95% CI)^a^	1.10	(1.02–1.18)	1	(Ref)
PDA requiring surgery				
Case/N	1,656/13,252	(12.5)	144/2703	(5.3)
RR (95% CI)^a^	1.65	(1.34–2.02)	1	(Ref)
Necrotizing enterocolitis				
Case/N	356/13,288	(2.7)	43/2705	(1.6)
RR (95% CI)^a^	1.22	(0.82–1.83)	1	(Ref)
Mortality				
Case/N	836/13,346	(6.3)	98/2701	(3.6)
RR (95% CI)^a^	1.31	(0.96–1.78)	1	(Ref)

Data are presented as the raw number (%) and adjusted RR (95% CI). ^a^Adjusted for maternal factors (facility level at birth, birth year, maternal age, hypertensive disorder of pregnancy, clinical chorioamnionitis, premature rupture of membrane, antenatal steroids, cesarean section, and non-reassuring fetal status) and neonatal factors (sex, gestational age, birth weight, small for gestational age, multiple births, Apgar score at 1 min, Apgar score at 5 min, placental transfusion, and respiratory distress syndrome). CI, confidence interval; CLD, chronic lung disease; IVH, intraventricular hemorrhage; PDA, patent ductus arteriosus; RR, risk ratio.

When stratified by gestational age (24–25 and 26–27 gestational weeks), the risks for severe IVH, any grade IVH, and PDA among eligible infants with DRI did not change substantially in either gestational age group (Table 4). However, the risks for CLD disappeared in the 24–25 gestational age group.

**Table 4:** Associations between delivery room intubation and neonatal morbidities and mortality, stratified by gestational age category.

	Delivery room intubation							
	24–25 weeks of gestational age				26–27 weeks of gestational age			
	Intubation		No intubation		Intubation		No intubation	
	(n = 5797)		(n = 541)		(n = 7570)		(n = 2173)	
Severe IVH (grade 3–4)								
Case/N	563/5797	(9.7)	21/541	(3.9)	343/7570	(4.5)	42/2173	(1.9)
RR (95% CI)^a^	2.35	(1.25–4.42)	1	(Ref)	1.68	(1.12–2.50)	1	(Ref)
IVH (any grade)								
Case/N	1654/5797	(28.5)	75/541	(13.9)	1,196/7570	(15.8)	175/2173	(8.1)
RR (95% CI)^a^	1.71	(1.30–2.24)	1	(Ref)	1.61	(1.33–1.95)	1	(Ref)
Periventricular leukomalacia								
Case/N	210/5769	(3.6)	11/539	(2.0)	300/7530	(4.0)	75/2168	(3.5)
RR (95% CI)^a^	1.56	(0.74–3.30)	1	(Ref)	1.24	(0.87–1.77)	1	(Ref)
CLD								
Case/N	2791/5131	(54.4)	228/506	(45.1)	2410/6997	(34.4)	511/2043	(25.0)
RR (95% CI)^a^	1.02	(0.92–1.13)	1	(Ref)	1.12	(1.02–1.23)	1	(Ref)
PDA requiring surgery								
Case/N	1,015/5745	(17.7)	62/538	(11.5)	641/7507	(8.5)	82/2165	(3.8)
RR (95% CI)^a^	1.42	(1.06–1.92)	1	(Ref)	1.76	(1.34–2.31)	1	(Ref)
Necrotizing enterocolitis								
Case/N	213/5760	(3.7)	15/539	(2.8)	143/7528	(1.9)	28/2,166	(1.3)
RR (95% CI)^a^	0.99	(0.52–1.87)	1	(Ref)	1.43	(0.86–2.38)	1	(Ref)
Mortality								
Case/N	525/5791	(9.1)	44/539	(8.2)	311/7555	(4.1)	54/2162	(2.5)
RR (95% CI)^a^	1.17	(0.74–1.85)	1	(Ref)	1.33	(0.88–2.01)	1	(Ref)

Data are presented as the raw number (%) and adjusted RR (95% CI). ^a^Adjusted for maternal factors (facility level at birth, birth year, maternal age, hypertensive disorder of pregnancy, clinical chorioamnionitis, premature rupture of membrane, antenatal steroids, cesarean section, and non-reassuring fetal status) and neonatal factors (sex, gestational age, birth weight, small for gestational age, multiple births, Apgar score at 1 min, Apgar score at 5 min, placental transfusion, and respiratory distress syndrome). CI, confidence interval; CLD, chronic lung disease; IVH, intraventricular hemorrhage; PDA, patent ductus arteriosus; RR, risk ratio.

Table 5 shows the associations between DRI and death or severe IVH among EPT infants without severe asphyxia. EPT infants who were intubated in the delivery room had a higher risk of death or severe IVH than those who were not. When stratified by gestational age (24–25 and 26–27 gestational weeks), we obtained similar findings.

**Table 5:** Associations between delivery room intubation and death or severe IVH among EPT infants without severe asphyxia.

	Delivery room intubation			
	Intubation		No intubation	
24–27 weeks of gestational age				
Death or severe IVH (grade 3–4)				
Case/N	1502/13,346	(11.3)	151/2703	(5.6)
RR (95%CI)^a^	1.47	(1.17–1.84)	1	(Ref)
24–25 weeks of gestational age				
Death or severe IVH (grade 3–4)				
Case/N	918/5791	(15.9)	59/539	(10.9)
RR (95%CI)^a^	1.48	(1.02–2.14)	1	(Ref)
26–27 weeks of gestational age				
Death or severe IVH (grade 3–4)				
Case/N	584/7555	(7.7)	92/2164	(4.3)
RR (95%CI)^a^	1.43	(1.07–1.91)	1	(Ref)

Data are presented as the raw number (%) and adjusted RR (95%CI). CI, confidence interval; EPT, extremely preterm; IVH, intraventricular hemorrhage; RR, risk ratio. ^a^Adjusted for maternal factors (facility level at birth, birth year, maternal age, maternal diabetes mellitus, preeclampsia, clinical chorioamnionitis, premature rupture of membrane, antenatal steroids, cesarean section, and non-reassuring fetal status) and neonatal factors (sex, gestational age, birth weight, small for gestational age, multiple births, Apgar score at 1 min, Apgar score at 5 min, and respiratory distress syndrome).

## Discussion

The present study examined the associations between DRI and severe IVH and other neonatal outcomes among EPT infants without severe asphyxia. EPT infants who were intubated in the delivery room were at higher risk for severe IVH, any grade IVH, CLD, and PDA requiring surgery than those who were not. When stratified by gestational age (i.e., 24–25 and 26–27 gestational weeks), the findings for severe IVH, any grade IVH, and PDA did not change substantially in either gestational age group. Furthermore, the sensitivity analysis showed an association between DRI and death or severe IVH.

The present study showed that EPT infants who were intubated in the delivery room had a higher risk of severe IVH and any grade IVH than those who were not. A retrospective cohort study in Canada showed that DRI was associated with higher odds of death and severe neurological injury compared with NICU intubation or no intubation among infants born at ≤ 28 gestational weeks^6^, which was consistent with our findings. A large retrospective cohort study in California showed that the differences in the results of DRI for severe IVH stratified by gestational age category (e.g., 24–25 gestational weeks, odds ratio 1.20, 95% CI 0.98–0.147; 26–27 gestational weeks, odds ratio 1.40, 95% CI 1.16–1.69)^5^. The risk for severe IVH in infants born at 24–25 gestational weeks with DRI in the present study may be related to differences in respiratory management policies in the delivery room, as described above^12^. Endotracheal intubation is associated with abnormal physiologic responses^27^, fluctuations in cerebral blood flow, and increased intracranial pressure^28^, which may increase the risk of IVH, especially in premature infants. Although systematic reviews of randomized controlled trials demonstrated that the risk for severe IVH did not change between invasive and non-invasive respiratory management in the delivery room^29–31^, a quality improvement project in the US showed that delivery room management intervention, including avoiding delivery room intubation, was associated with reductions in IVH among infants born at < 30 gestational weeks^32^. The results of the present study do not inherently indicate a causal relationship between DRI and severe IVH, primarily due to confounding by indication. However, DRI may serve as an indicator of the severity of the underlying disease, thereby facilitating the identification of neonates who are more susceptible to severe IVH.

In this study, EPT infants who were intubated in the delivery room had an increased risk of PDA requiring surgery compared with those who were not. A retrospective cohort study in Taiwan demonstrated that very-low-birth-weight infants without endotracheal intubation were likely to have spontaneous PDA closure in the first week following birth^33^. Furthermore, secondary analysis from a randomized trial comparing nasal high-flow with continuous positive airway pressure as primary respiratory support in Australia showed that among infants born at 28–32 gestational weeks, infants who were intubated were more likely to have PDA requiring treatment^34^. These results are consistent with the findings of the present study, suggesting an adverse effect of the respiratory condition requiring endotracheal intubation on PDA. Conversely, we did not find that infants who were intubated in the delivery room had a better prognosis for other neonatal outcomes than those who were not intubated.

The trend in DRI rates among EPT infants in Japan was not consistent with the trend seen in other countries such as the US^5, 8–10^. For instance, the rate of DRI among infants born at 22–28 weeks of gestation in the US decreased from 80% in 1993 to 65% in 2012, while the incidence of severe IVH declined from 19 to 13% over the same period^8^. Moreover, in Japan, the rate of DRI among infants born at 22–27 gestational weeks (90.8% in 2015–2019, Supplementary Table S1 online) was higher than that in the US (71.0% in 2013–2018)^4^. The International Network of Evaluating Outcomes of Neonates found that most NICUs in Japan use an invasive approach for EPT infants^13^, despite evidence from systematic reviews suggesting that non-invasive respiratory management is more effective in reducing the risk of death or CLD^29–31^. The prevalence of invasive respiratory management among Japanese neonatologists may stem from concerns that a non-invasive approach in the delivery room could increase the risk of mortality and IVH among EPT infants^12^. The decreasing trend of severe IVH in Japan may be due to the widespread use of antenatal steroids rather than increased DRI.

The main strength of the present study was the large sample size with precise perinatal data from a national database in Japan, which allowed us to estimate the association between DRI and severe IVH, even in a country with a low incidence of severe IVH. However, this study had several limitations. First, this was a retrospective observational study. Therefore, additional unmeasured confounding factors might have affected the results. For example, there were no data on indications for DRI, administration of surfactant in the delivery room, or the severity of EPT infants (e.g., Clinical Risk Index for Babies score)^35^. The differences in morbidities between EPT infants with DRI and those without DRI may be due to the differences in the severity of the two groups. Clinicians might have not intubated vigorous infants, which may cause reverse causation. Second, we did not have data on the implementation of intubate-surfactant-extubate or less-invasive-surfactant-administration approaches for EPT infants. However, as mentioned above, the International Network of Evaluating Outcomes of Neonates found that most NICUs in Japan used invasive respiratory management with all EPT infants, and intubate-surfactant-extubate and less-invasive-surfactant-administration approaches were less common than in other countries^13^. Third, we could not exclude severely asphyxiated infants from the intubation group because emergent intubation before 5 min of age affected the Apgar score at 5 min, which may overestimate the adverse effect of DRI. Fourth, there was no information on the details of DRI, such as the number of attempts, experience of operators, or use of premedication. Fifth, the timing of IVH occurrence was not available in the NRNJ database, although this study focused on the relationship between early postnatal intubation and IVH.

In conclusion, the present study shows that DRI was associated with severe IVH among EPT infants in a country with low baseline rates of severe IVH and high DRI rates. Further prospective studies are needed to determine whether avoiding intubation in the delivery room could reduce the incidence of severe intraventricular hemorrhage even in a country with high DRI rates.

### Supplementary Information

Supplementary Information 1.

Supplementary Table S1.

Supplementary Table S2.

Supplementary Information 4.

## Data Availability

The data supporting the results of this study are available from the Neonatal Research Network of Japan, but there are restrictions on the availability of these data, which were used under license for the current study and are therefore not publicly available. However, the data are available from the corresponding author upon reasonable request and with the permission of the Neonatal Research Network of Japan.
