# Supplementary material for: Delivery room intubation and severe intraventricular hemorrhage in extremely preterm infants without low Apgar scores: A Japanese retrospective cohort study
Source: Sci Rep. 2023 Sep 11;13:14990. doi: 10.1038/s41598-023-41010-x (PMC10495461; doi:10.1038/s41598-023-41010-x)
Supplement: Supplementary file 1 — Supplementary Information 1. [file 41598_2023_41010_MOESM1_ESM.pdf]

The institutions enrolled in the present study of the NRNJ are: Sapporo City Hospital, Asahikawa Kosei Hospital, Engaru-Kosei Hospital, Kushiro Red Cross Hospital, Obihiro Kosei Hospital, Tenshi Hospital, JCHO Hokkaido Hospital, NTT East Sapporo Hospital, Nikko Memorial Hospital, Nayoro City Hospital, Sapporo Medical University, Asahikawa Medical University, Aomori Prefectural Central Hospital, Iwate Medical University, Iwate Prefectural Ofunato Hospital, Iwate Prefectural Kuji Hospital, Iwate Prefectural Ninohe Hospital, Sendai Red Cross Hospital, Tohoku University, Akita Red Cross Hospital, Akita University, Tsuruoka City Shonai Hospital, Yamagata University, Yamagata Prefectural Central Hospital, Fukushima Medical University, Takeda General Hospital, National Fukushima Hospital, Tsukuba University, Tsuchiura Kyodo Hospital, Ibaraki Children’s Hospital, Dokkyo Medical University, Jichi Medical University, Ashikaga Red Cross Hospital, Gunma Children’s Medical Center, Kiryu Kosei General Hospital, Ohta General Hospital, Gunma University, Saitama Medical University, Saitama Prefectural Children's Medical Center, National Nishisaitama Central Hospital, Saitama Medical University Medical Center, Kawaguchi City Medical Center, Jichi Medical University Saitama Medical Center, Asahi Central Hospital, Chiba City Kaihin Hospital, Kameda General Hospital, Tokyo Women’s Medical University Yachiyo Medical Center, Juntendo University Urayasu Hospital, Narita Red Cross Hospital, Tokyo Metropolitan Children's Medical Center, Tokyo Women's Medical University, Aiiku Hospital, Nihon University, Center Hospital of the National Center for Global Health and Medicine, Tokyo Medical University, Teikyo University, Showa University, Japan Red Cross Hospital, National Center for Child Health and Development, Tokyo Metropolitan Otsuka Hospital, Tokyo University, Toho University, Tokyo Metropolitan Bokuto Hospital, Tokyo Jikei Medical University, Tokyo Medical and Dental

University, Saint Luke’s Hospital, Juntendo University, San-Ikukai Hospital, Tokyo Katsushika Red Cross Perinatal Center, Yokohama Rosai Hospital, Yokohama City University Medical Center, St. Marianna Medical University, Kanagawa Children's Medical Center, Tokai University, Kitasato University, Yokosuka Kyosai Hospital, Odawara City Hospital, Nippon Medical School Musashi Kosugi Hospital, Yokohama City Hospital, Saiseikai Eastern Yokohama Hospital, Yokohama Medical Center, Yamanashi Prefectural Central Hospital, Nagano Children’s Hospital, Shinshu University, Iida City Hospital, Shinshu Ueda Medical Center, Saku General Hospital, Niigata University, Niigata Central Hospital, Niigata City Hospital, Nagaoka Red Cross Hospital, Kouseiren Takaoka Hospital, Toyama Prefectural Central Hospital, Toyama University, Ishikawa Prefectural Central Hospital, Kanazawa Medical University, Kanazawa Medical Center, Fukui Prefectural Hospital, Fukui University, Gifu Prefectural Medical Center, Ogaki City Hospital, Nagara Medical Center, Takayama Red Cross Hospital, Seirei Hamamatsu Hospital, Shizuoka Saiseikai Hospital, Shizuoka Children’s Hospital, Hamamatsu Medical University, Numazu City Hospital, Yaizu City Hospital, Fujieda City Hospital, Nagoya Red Cross Daini Hospital, Nagoya University, Nagoya Red Cross Daiici Hospital, Toyohashi City Hospital, Nagoya City Seibu Medical Center, Fujita Medical University, Anjo Kosei Hospital, Tosei General Hospital, Komaki City Hospital, Toyota Memorial Hospital, Okazaki City Hospital, Handa City Hospital, Konan Kosei Hospital, Nagoya City University, Aichi Medical University, Mie Central Medical Center, Ise Red Cross Hospital, Yokkaichi City Hospital, Otsu Red Cross Hospital, Shiga Medical University, Nagahama Red Cross Hospital, Uji Tokushukai Hospital, Japan Baptist Hospital, Kyoto University, Kyoto Red Cross Daiichi Hospital, Maizuru Medical Center, Fukuchiyama City Hospital,

Kyoto Prefectural University of Medicine, Kyoto City Hospital, Mitsubishi Kyoto Hospital, Yodogawa Christian Hospital, Osaka Women's and Children's Hospital, Osaka University, Takatsuki General Hospital, Kansai Medical University, Osaka City General Hospital, Osaka City Sumiyoshi Hospital, Aizenbashi Hospital, Toyonaka City Hospital, National Cerebral and Cardiovascular Center, Kitano Hospital, Saiseikai Suita Hospital, Chifune Hospital, Bell Land General Hospital, Rinku General Hospital, Osaka Red Cross Hospital, Yao City Hospital, Hannan Central Hospital, Osaka General Medical Center, Osaka City University, Kobe Children's Hospital, Kobe University, Kakogawa City Hospital, Saiseikai Hyogo Hospital, Kobe City Medical Center General Hospital, Hyogo Medical University, Himeji Red Cross Hospital, Toyooka General Hospital, Hyogo Prefectural Awaji Medical Center, Nara Medical University, Wakayama Medical University, Tottori Prefectural Central Hospital, Tottori University, Shimane Prefectural Central Hospital, Matsue Red Cross Hospital, Kurashiki Central Hospital, Tsuyama Central Hospital, Kawasaki Medical University, Okayama Medical Center, Okayama Red Cross Hospital, Hiroshima City Central Hospital, Hiroshima Prefectural Hospital, Hiroshima University, Tsuchiya General Hospital, Kure Medical Center, Yamaguchi University, Yamaguchi Prefectural Grand Medical Center, Tokushima University, Tokushima City Hospital, Tokushima Prefectural Central Hospital, Kagawa University, Shikoku Medical Center for Children and Adults, Matsuyama Red Cross Hospital, Ehime Prefectural Central Hospital, Kochi Health Science Center, Saint Maria Hospital, Kyushu Medical Center, Kurume University, Kitakyushu City Hospital, University of Occupational and Environmental Health Japan, Fukuoka University, Kyushu University, Iizuka Hospital, Kokura Medical Center, Fukuoka City Children's Hospital, Saga Hospital, Nagasaki University, Nagasaki Medical Center, Sasebo City Hospital,

Kumamoto City Hospital, Kumamoto University, Oita Prefectural Hospital, Almeida Memorial Hospital, Nakatsu City Hospital, Miyazaki University, Miyakonojo Medical Center, Kagoshima City Hospital, Imakyure General Hospital, Okinawa Prefectural Nanbu Medical Center & Children’s Medical Center, Okinawa Chubu Hospital, Naha City Hospital, and Okinawa Red Cross Hospital.
