# Supplementary material for: Delivery room intubation and severe intraventricular hemorrhage in extremely preterm infants without low Apgar scores: A Japanese retrospective cohort study
Source: Sci Rep. 2023 Sep 11;13:14990. doi: 10.1038/s41598-023-41010-x (PMC10495461; doi:10.1038/s41598-023-41010-x)
Supplement: Supplementary file 2 — Supplementary Table S1. [file 41598_2023_41010_MOESM2_ESM.pdf]

Supplementary Table S1. Trends in treatment and morbidities of extremely preterm infants (22-27 weeks of gestational age) including those with low Apgar scores in the Neonatal Research Network of Japan.

	Birth Year					
	2003-2008 (n=6,799)		2009-2014 (n=9,941)		2015-2019 (n=6,428)	
Delivery room intubation	5,679	(83.5)	8,683	(87.3)	5,838	(90.8)
22-25 weeks of gestational age	3,264/3,538	(92.3)	4,756/5,071	(93.8)	3,254/3,389	(96.0)
26-27 weeks of gestational age	2,415/3,261	(74.1)	3,927/4,870	(80.6)	2,584/3,039	(85.0)
Antenatal steroids	2,959	(43.5)	5,923	(60.4)	4,418	(69.6)
Cesarean section	4,722	(70.2)	7,505	(75.7)	5,113	(80.2)
Respiratory distress syndrome	4,949	(72.8)	7,856	(79.5)	5,366	(83.9)
Surfactant administration during hospitalization	5,062	(74.5)	8,153	(83.0)	5,596	(88.2)
Mortality	1,108	(16.3)	1,004	(10.1)	552	(8.6)
Severe IVH (grade 3-4)	693	(10.2)	875	(8.8)	531	(8.3)
IVH (any grade)	1,740	(25.6)	2,406	(24.2)	1,596	(24.8)
Chronic lung disease	1,952	(34.1)	4,102	(47.5)	3,013	(54.0)

Values are n (%).
IVH, intraventricular hemorrhage.
