# Supplementary material for: Delivery room intubation and severe intraventricular hemorrhage in extremely preterm infants without low Apgar scores: A Japanese retrospective cohort study
Source: Sci Rep. 2023 Sep 11;13:14990. doi: 10.1038/s41598-023-41010-x (PMC10495461; doi:10.1038/s41598-023-41010-x)
Supplement: Supplementary file 3 — Supplementary Table S2. [file 41598_2023_41010_MOESM3_ESM.pdf]

Supplementary Table S2. Baseline characteristics, stratified by gestational age category

	Delivery room intubation							
	24-25 weeks of gestational age				26-27 weeks of gestational age			
	Intubation (n=5,797)		No intubation (n=541)		Intubation (n=7,570)		No intubation (n=2,173)	
Characteristics of infants								
Mean gestational age (SD), weeks	25.0	(0.6)	25.1	(0.6)	26.9	(0.6)	27.1	(0.6)
24 or 26 weeks	2,632	(45.4)	182	(33.6)	3,691	(48.8)	757	(34.8)
25 or 27 weeks	3,165	(54.6)	359	(66.4)	3,879	(51.2)	1,416	(65.2)
Mean birth weight (SD), g	691	(131)	715	(124)	871	(188)	925	(175)
<500 g	494	(8.5)	29	(5.4)	323	(4.3)	24	(1.1)
500-749 g	3,384	(58.4)	306	(56.6)	1,490	(19.7)	298	(13.7)
750-999 g	1,881	(32.4)	199	(36.8)	3,958	(52.3)	1,126	(51.8)
≥1000 g	38	(0.7)	7	(1.3)	1,799	(23.8)	725	(33.4)
Small for gestational age	991	(17.1)	67	(12.4)	2,016	(26.6)	410	(18.9)
Sex, male	3,013	(52.0)	279	(51.7)	4,011	(53.0)	1,167	(53.7)
Multiple births	963	(16.6)	70	(12.9)	1,788	(23.6)	400	(18.4)
Facility level at birth								
Tertiary	4,999	(86.2)	501	(92.6)	6,210	(82.0)	1,895	(87.2)
Secondary	715	(12.3)	40	(7.4)	1,230	(16.2)	262	(12.1)
Others	83	(1.4)	0	(0.0)	130	(1.7)	16	(0.7)
Birth year								
2003-2009	1,728	(29.8)	202	(37.3)	2,060	(27.2)	829	(38.2)
2010-2014	2,462	(42.5)	248	(45.8)	3,364	(44.4)	912	(42.0)
2015-2019	1,607	(27.7)	91	(16.8)	2,146	(28.3)	432	(19.9)
Median Apgar score at 1min (25-75%tile)	4	(3-5)	6	(4-7)	5	(3-6)	6	(5-8)
2 to 3	2,055	(35.5)	79	(15.4)	2,006	(26.6)	208	(9.7)
4 to 7	3,484	(60.2)	342	(66.8)	5,002	(66.3)	1,342	(62.9)
8 to 10	248	(4.3)	91	(17.8)	541	(7.2)	584	(27.4)
Median Apgar score at 5min (25-75%tile)	7	(6-8)	8	(7-9)	7	(6-8)	8	(7-9)
4 to 7	3,887	(67.9)	205	(40.5)	4,363	(58.4)	567	(26.7)

8 to 10	1,840	(32.1)	301	(59.5)	3,104	(41.6)	1,558	(73.3)
Placental transfusion	1,822	(31.4)	110	(20.3)	2,171	(28.7)	383	(17.6)
Respiratory distress syndrome	4,692	(81.3)	280	(51.9)	6,104	(81.0)	1,194	(55.1)
Surfactant administration during hospitalization	4,957	(86.4)	288	(53.5)	6,223	(82.9)	1,129	(52.1)
Maternal characteristics								
Maternal age, years								
<25	614	(10.8)	57	(11.4)	751	(10.1)	225	(10.7)
25-29	1,338	(23.4)	123	(24.6)	1,713	(23.0)	485	(23.1)
30-34	1,972	(34.6)	182	(36.4)	2,619	(35.1)	736	(35.0)
≥35	1,782	(31.2)	138	(27.6)	2,377	(31.9)	655	(31.2)
Antenatal steroids	3,566	(62.0)	338	(62.9)	4,536	(60.5)	1,344	(62.5)
Cesarean section	4,577	(79.1)	369	(68.5)	6,230	(82.6)	1,628	(75.1)
Non-reassuring fetal status	1,273	(22.5)	90	(16.8)	1,862	(25.2)	404	(19.0)
Premature rupture of membranes	2,364	(41.0)	252	(46.8)	2,629	(34.9)	856	(39.6)
Clinical chorioamnionitis	1,864	(32.9)	158	(30.5)	1,742	(23.6)	497	(23.7)
Hypertensive disorder of pregnancy	571	(9.9)	37	(6.9)	1,169	(15.5)	280	(12.9)

Values are n (%) unless otherwise indicated.
